# Marital status and abortion among young women in Rupandehi, Nepal

**DOI:** 10.1186/s12905-015-0175-4

**Published:** 2015-02-22

**Authors:** Kathryn L Andersen, Ram Chandra Khanal, Alexandra Teixeira, Shailes Neupane, Sharad Sharma, Valerie N Acre, Maria F Gallo

**Affiliations:** Ipas, 300 Market St., Suite 200, Chapel Hill, NC 27516 USA; Ipas Nepal, PO Box 11621, , Tewa Tower, 3rd Floor, Teku, Kathmandu, Nepal; Valley Research Group, PO Box 4112, Kathmandu, Nepal; Division of Epidemiology, The Ohio State University, College of Public Health, 324 Cunz Hall 1841 Neil Avenue, Columbus, OH 43210 USA

**Keywords:** Adolescents, Induced abortion, Nepal, Pregnancy

## Abstract

**Background:**

Despite liberalization of the Nepal abortion law, young women continue to experience barriers to safe abortion services. We hypothesize that marital status may differentially impact such barriers, given the societal context of Nepal.

**Methods:**

We evaluated differences in reproductive knowledge and attitudes by marital status with a probability-based, cross-sectional survey of young women in Rupandehi district, Nepal. Participants (N = 600) were surveyed in 2012 on demographics, romantic experiences, media habits, reproductive information, and abortion knowledge and attitudes. We used logistic regression to assess differences by marital status, controlling for age.

**Results:**

Participants, who comprised never-married (54%) and ever-married women (45%), reported good access to basic reproductive health and abortion information. Social desirability bias might have prevented reporting of premarital romantic and sexual activity given that participants reported more premarital activities for their friends than for themselves. Only 45% knew that abortion was legal, and fewer ever-married women were aware of abortion legality. Never-married women expected more negative responses from having an abortion than ever-married women.

**Conclusions:**

Findings highlight the need for providing sexual and reproductive health care information and services to young women regardless of marital status.

## Background

Since legalizing abortion in 2002 [1], Nepal has made striking progress in rolling out induced abortion services, establishing comprehensive abortion care at public-sector facilities, and providing safe abortion care services to >500,000 Nepali women [2]. The relatively liberal law, which allows for legal termination of pregnancy up to 12 weeks of gestation–or up to 18 weeks in cases of result of rape or incest or at any time if medically indicated–appears to have contributed to an overall national decline in maternal mortality [3]. However, unsafe abortion remains a health concern. Abortion accounted for an estimated 14% of maternal deaths at health care facilities in 2008–2009 [4], which underscores that even in settings where abortion is legal, women may resort to unsafe procedures. In general, barriers to accessing safe abortion care can include negative provider attitudes, fear of repercussion, lack of access to comprehensive sexuality education, limited financial resources, cost of care, transportation, third-party involvement laws, and concerns over privacy and confidentiality [5].

Adolescents, in particular, often face challenges in accessing abortion. Young women who obtain abortion care tend to access it later in pregnancy than older women [6] and are more likely to delay seeking help for abortion-related complications [7]. These delays likely are attributable, at least in part, to stigma surrounding adolescent sexuality [8-10]. Many youth lack the negotiation and decision-making skills necessary for abstaining from unsafe sexual practices [11]. In Nepal, only 4.2% of adolescents report using a modern form of contraception [12], and youth describe embarrassment from discussing sexual health with parents, relatives and senior community members [13]. Adolescents also perceive sexual health service providers as judgmental and raise concerns about lack of confidentiality.

Less research has been conducted on the relationship between marital status and abortion. The patriarchal and patrilineal nature of society in Nepal translates into a strong preference for sons [14,15], which could influence women’s decision-making about and access to abortion. Having a male offspring is considered crucial for continuing the family lineage, providing financial support to parents as they age, and carrying out other roles (e.g., lighting funeral pyres) while female children are often viewed as a financial burden (e.g., because of dowry costs). Married women in Nepal perceive great pressure from their husband and parents-in-law to bear a son [16], and abortion for sex selection has emerged as a concern [17,18]. Despite the illegality of abortion for this purpose in Nepal [1], we hypothesize that the intense pressure that married women are under to have a male child could contribute to a greater acceptance of abortion–regardless of the reason in individual cases–for married women compared to unmarried women.

Despite cultural norms against premarital sexual activity in Nepal [19], the practice appears to be common [20,21]. Social proscriptions against sex outside of marriage, though, could limit unmarried women’s access to contraception or safe abortion services. For example, government hospitals and some pharmacies distribute condoms only to married individuals [22], and their purchase by unmarried women is considered culturally unacceptable. A study conducted at the largest women’s hospital in Nepal found that most induced abortions were performed on married women [23]. This disparity by marital status could reflect a greater need for abortion services among married women or could result from unmarried women being less likely to access safe services. We hypothesized that regardless of their age, unmarried women in a patriarchal society such as Nepal might face different barriers to obtaining safe abortion care than their married counterparts.

## Methods

We conducted a cross-sectional survey during April and May 2012 using a probability-based household sample of 600 women aged 16–24 years in Rupandehi district, Nepal. The district was selected for its strong community partners (for planning future interventions), its geographic accessibility, and its diverse representation of the nation’s castes and ethnicities and major religions, namely, Hinduism, Muslim and Buddhism. One of 75 districts in Nepal, Rupandehi district is located in the Lumbini Zone in the Western Development Region and is rapidly urbanizing, with a population growth rate of 3.05 percent in [24].

Trained female interviewers administered a survey to collect information on participant demographics, romantic and sexual experiences, media habits, sources and types of reproductive information, and abortion knowledge and attitudes. The survey was developed in English and translated into Nepali before being pretested with approximately 20 young women from a nearby geographic area, who were expected to be similar to the target population. All interviews were conducted in a private setting. Only women who provided written consent were eligible for participation. The National Health Research Council (NHRC) in Nepal and Allendale institutional review board in the U.S. approved the protocol.

### Sampling

The sample was selected with a two-stage cluster sampling design. In the first stage, the clusters (wards) were identified using the probability proportional to size (PPS) method among all wards within the intervention area (using ward populations from 2001 census data) [25]. The sample was conducted from 37 of Rupandehi’s 71 main socio-political units (including 69 village development committees (VDCs) and two municipalities, which are each subdivided by population size into approximately nine wards. After listing all intervention wards in alphabetic order by VDC, municipality and ward name, we used the PPS method to select 30 wards.

In the second stage of sampling, we randomly selected 600 respondents from households within each sampled ward. First, we consulted with key informants from the selected wards to create lists of household heads within each selected ward. Then, we used simple random sampling (using a random start and sampling interval) to select 20 households from each list. Using simple sketched maps of the selected wards to locate households, interviewers administered a screening questionnaire to the head or knowledgeable person of the sampled household to determine the residence of any women 16–24 years of age. If multiple women meeting the age criterion resided in the household, only one was randomly selected for participation using a lottery technique. If the selected respondent was unavailable after three visits or if she declined to consent, the nearest neighboring household was substituted.

### Analysis

We report simple descriptive statistics for the factors assessed. For socio-demographic characteristics and reproductive history, we assessed differences between never- married and ever-married women using chi-squared tests and t tests. We used logistic regression models adjusted for age (in years) in order to identify potential differences by marital status in media habits, type and sources of information on abortion and contraception, and abortion knowledge and perceptions.

## Results

### Study sample

We visited a total of 805 households, of which 611 households had at least one woman 16–24 years of age. In 11 of the households, no woman was available to be interviewed due to not being present at the time of the interview (n = 6), being sick or physically weak (n = 3) or declining participation (n = 2). Thus, the analysis is based on interviews with 600 participants.

About 54% of the participants were never married while the remainder were currently married (45%) or were separated, divorced or widowed (1%). Mean age at marriage was 16.4 years (standard deviation [SD], 2.6; range, 8–23). Never- and ever-married women differed in their demographic characteristics and reproductive history (Table 1). The mean age of respondents was 19.2 years (SD, 2.7) with a younger mean age among those who had never been married compared with that of ever-married women (17.8 and 20.9 years, respectively; p-value <0.01). More never-married women (67%) had completed ≥6 grades of education than their ever-married counterparts (33%; p = 0.01). Few of the never-married women reported sexual debut and contraception use, and none reported having been pregnant or having had an abortion.

**Table 1:** **Socio-demographic characteristics and reproductive history, overall and by marital status, Rupandehi district, 2012**

	**Total**		**Never married**		**Ever married**		p*
	**(N = 600)**		**(N = 325)**		**(N = 275)**		
Age, median (range)	19	(16–24)	17	(16–24)	21	(16–24)	<0.01
	**No.**	**(%)**	**No.**	**(%)**	**No.**	**(%)**	
Education completed							
< Grade 6	203	(33.8)	58	(28.6)	145	(71.4)	<0.01
≥ Grade 6	397	(66.2)	267	(67.3)	130	(32.8)	
Caste or ethnicity							
Brahmin/Chhetri	106	(17.7)	68	(64.2)	38	(35.9)	
Terai/Madhesi/Other	241	(40.2)	111	(46.1)	130	(53.9)	
Dalit	55	(9.2)	24	(43.6)	31	(56.4)	
Newar	5	(0.8)	3	(60.0)	2	(40.0)	
Janajati	153	(25.5)	100	(65.4)	53	(34.6)	
Muslim	40	(6.7)	19	(47.5)	21	(52.5)	
Worked in past year (cash or in-kind)							
Yes	76	(12.7)	50	(15.4)	26	(9.5)	0.03
No	524	(87.3)	275	(84.6)	249	(90.6)	
Ever had sex							
Yes	282	(47.0)	7	(2.2)	275	(100.0)	<0.01
No	318	(53.0)	318	(97.9)	0	(0.0)	
Ever used contraception							
Yes	137	(22.8)	5	(1.5)	132	(48.0)	<0.01
No	463	(77.2)	320	(98.5)	143	(52.0)	
Gravidity							
0	400	(66.7)	325	(100.0)	75	(27.3)	<0.01
≥1	200	(33.3)	0	(0.0)	325	(72.7)	
Abortion history							
0	581	(96.8)	325	(100.0)	256	(93.1)	0.01
≥1	19	(3.2)	0	(0.0)	19	(6.9)	

*P-value from unadjusted chi-squared test or t test for difference by marital status.

### Romantic experiences

**Figure 1:**
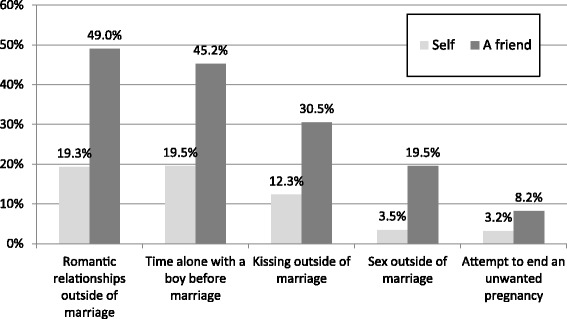
**Romantic and sexual experiences involving self or a friend, Rupandehi district, 2012.**

Few participants reported having had romantic experiences (i.e., relationships, time alone with a boy, kissing, or sex) outside of the context of marriage or attempting to end an unwanted pregnancy (Figure 1). However, when asked about the experiences of their *friends*, participants gave more accounts of each activity, with 49% of participants reporting having a friend with a romantic relationship outside of marriage, 20% having a friend have sex outside of marriage and 8% having a friend attempt abortion.

### Media habits

More than half of respondents (53%) reported viewing television daily, with another 24% watching television at least once per week. Furthermore, 38% and 23% of women listened to the radio either daily or at least once per week, respectively. Print media were relatively popular among participants, with 40% reporting newspaper or magazine use. Fewer women (10%) reported any internet use. Among those using the internet, most did so in their own homes (68%) and many accessed the social website Facebook (86%). Reported attendance at youth or school clubs was low (5%). Notably, a higher proportion of never-married participants engaged with media sources than ever-married participants, particularly with regard to daily television (60% vs. 45%, respectively; p < 0.01), daily radio (43% vs. 32%, respectively; p < 0.01) and print media (52% vs. 25%, respectively; p < 0.01).

### Types and sources of reproductive information

Most participants reported receiving information about contraceptive methods (89%) and sources of services (85%) in the past year (Table 2). Fewer were informed on their costs (28%), directions on use (11%) and side effects (11%). Regarding abortion, while most women reported receiving information in the past year on where to obtain a safe abortion (76%), fewer were informed about abortion methods (52%), costs (31%), and possible complications (43%). Most participants were able to identify medical abortion (82%) and dilation and curettage (69%) as methods of abortion while few (8%) had heard of manual vacuum aspiration (MVA). Marital status did not appear to be related to the type of contraceptive information received in the past year. In contrast, married women were more likely to report receiving information on abortion costs (p = <0.01), possible complications (p = 0.02), legality (p = 0.02) and awareness of abortion methods (p = 0.02) than never-married women after controlling for age.

**Table 2:** **Information received and awareness of methods, overall and by marital status, Rupandehi district, 2012**

	**Total**		**Never married**		**Ever married**		
	**(N = 600)**		**(N = 325)**		**(N = 275)**		
	**No.**	**(%)**	**No.**	**(%)**	**No.**	**(%)**	p*
Contraception information received in past year							
Methods	535	(89.2)	290	(89.2)	245	(89.1)	0.44
Sources	512	(85.3)	278	(85.5)	234	(85.1)	0.45
Costs	131	(27.5)	66	(20.3)	65	(23.6)	0.50
Directions on use	165	(11.0)	92	(28.3)	73	(26.6)	0.38
Possible side effects	66	(11.0)	40	(12.3)	26	(9.5)	0.20
Abortion information received in past year							
Methods	311	(51.8)	165	(50.8)	146	(53.1)	0.61
Sources for safe abortion	458	(76.3)	249	(76.6)	209	(76.0)	0.23
Costs	184	(30.7)	75	(23.1)	109	(39.6)	<0.01
Possible complications	255	(42.5)	143	(44.0)	112	(40.7)	0.02
Stories of other women’s induced abortions	136	(22.7)	80	(24.6)	56	(20.4)	0.13
Abortion-related mortality	102	(17.0)	60	(18.5)	42	(15.3)	0.08
Legality of abortion	75	(12.5)	49	(15.1)	26	(9.5)	0.02
Aware of abortion methods							
No methods	74	(12.3)	35	(10.8)	39	(14.2)	
Yes, any method	526	(87.7)	290	(89.2)	236	(85.8)	0.02
Medication abortion	494	(82.3)	270	(83.1)	224	(81.5)	
Manual vacuum aspiration	42	(7.0)	25	(7.7)	17	(6.2)	
Dilation & curettage	412	(68.7)	227	(69.9)	185	(67.3)	

*P-value for difference by marital status from logistic regression analysis adjusted for age.

**Figure 2:**
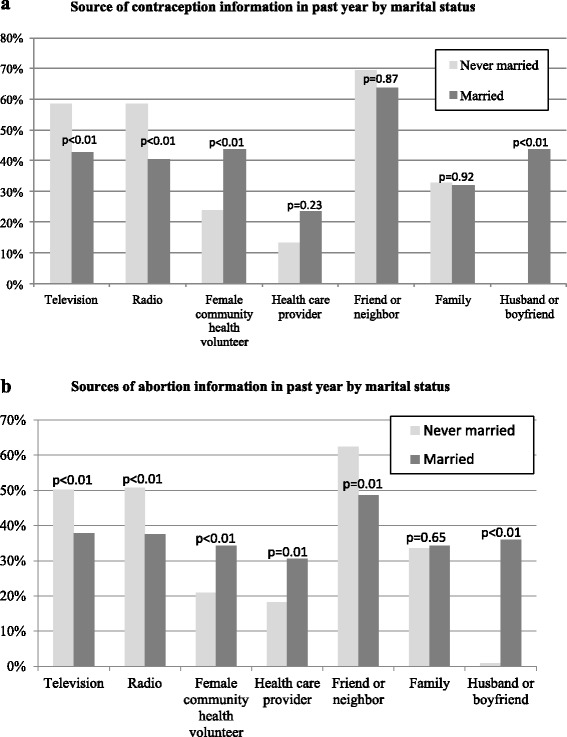
**Source of information in past year by marital status, Rupandehi district, 2012.** P-values for differences by marital status from logistic regression analysis adjusted for age. **a**. Contraceptive Information. **b**. Abortion Information.

Women received reproductive-health information from a variety of sources (Figure 2a and b). Not surprisingly, the most popular source of information on contraception and abortion was an interpersonal one: a friend or neighbor (67% and 56%, respectively). Television and radio also were common sources of information on contraception (52% and 50%, respectively) and, to a lesser extent, on abortion (45% and 27%, respectively). Female community health volunteers (FCHVs) were the most frequently cited among the professional and community sources for information on contraception (33%) and abortion (24%). Sources of information differed by marital status. For example, never-married participants were more likely to rely on television or radio for both contraception and abortion information whereas married women were more likely to receive information from FCHVs and health care providers.

### Abortion knowledge and attitudes

**Figure 3:**
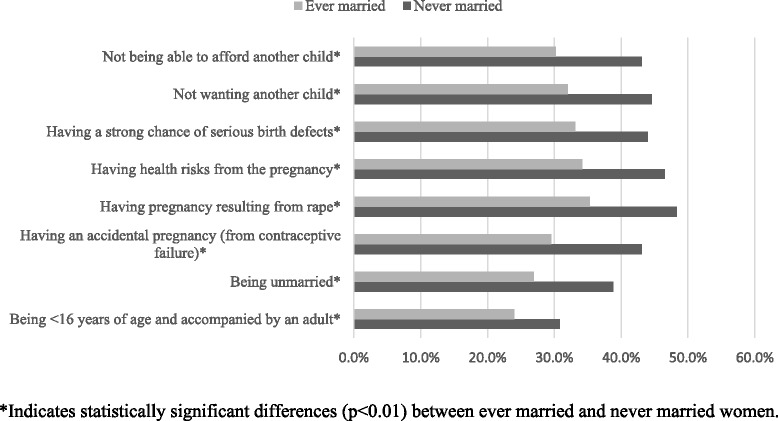
**Knowledge of factors that affect abortion legality in Nepal by marital status, Rupandehi district, 2012.**

Participants had low levels of knowledge about the legal status of abortion in Nepal: only 45% (N = 271) of respondents knew that termination of pregnancy was legal. Furthermore, the details of the law were not well known. For example, only 28% of women knew that 16 was the legal age of consent for abortion (i.e., younger women are required to be accompanied by a legal guardian). Only 33% of participants knew that being unmarried did not automatically make abortion illegal and only 37% knew that having an unintended pregnancies as the result of contraceptive failure does not preclude women from accessing legal abortions. Ever-married women consistently were less informed than never-married women even after controlling for age (Figure 3).

Respondents were asked whether they agreed or disagreed with a series of statements related to abortion practices (Table 3). Opinions were favorable for a number of important items such as support if friends needed an abortion (86%); feeling comfortable in talking with health care providers about abortion (74%); and feeling comfortable initiating talks with my friends about abortion-related issues (70%). However, participants believed that abortion costs and acceptability differ by the marital status of the woman involved. Most respondents believed that doctors usually charge more for an abortion for unmarried women (68%) and that abortion is more acceptable for married women (47%) than for unmarried women (16%) in their communities.

**Table 3:** **Abortion-related perceptions, overall and by marital status, Rupandehi district, 2012**

	**Total**		**Never married**		**Ever married**		P*
	**(N = 600)**		**(N = 325)**		**(N = 275)**		
**Agrees with statement**	**No.**	**(%)**	**No.**	**(%)**	**No.**	**(%)**	
If I needed an abortion and a facility were available in my village development committee, I would go there	411	(68.5)	208	(64.0)	203	(73.8)	0.09
I have information about abortion methods and providers near me	382	(63.7)	203	(62.5)	179	(65.1)	0.52
Doctors usually charge unmarried women more for an abortion	406	(67.7)	219	(67.4)	187	(68.0)	0.87
Abortions among unmarried women generally are considered acceptable in this community	93	(15.5)	38	(11.7)	55	(20.0)	0.08
Abortions among married women generally are considered acceptable in this community	282	(47.0)	152	(47.8)	130	(47.3)	0.36
Talking about abortion-related issues is common among my friends	404	(67.3)	226	(69.5)	178	(64.7)	0.01
If I were to have an abortion, my friends and relatives would treat me badly	380	(63.3)	228	(70.2)	152	(55.3)	<0.01
If I were to go for an abortion, I imagine that the health care workers would treat me kindly	269	(44.8)	121	(37.2)	148	(53.8)	<0.01
I feel comfortable talking to a medical doctor or nurse about abortion-related issues	445	(74.2)	241	(74.2)	204	(74.2)	0.14
I feel comfortable initiating talks with my friends about abortion-related issues	422	(70.3)	237	(72.9)	185	(67.3)	<0.01
If a friend of mine wanted an abortion, I feel confident that I could help her find this service	464	(77.3)	262	(80.6)	202	(73.5)	<0.01
I can say the words “vagina” and “penis” without feeling embarrassed	90	(15.0)	59	(18.2)	31	(11.3)	<0.01
I am able to say “no” if I do not feel like having sex	459	(76.5)	259	(76.7)	200	(72.7)	0.09
If a friend told me that she needed an abortion, I would support her	515	(85.8)	287	(88.3)	228	(82.9)	0.02

*P-value for difference by marital status from logistic regression analysis adjusted for age.

Furthermore, never-married women appeared more comfortable with the idea of abortion than ever-married women. More never-married women than their ever-married counterparts reported commonly talking about abortion-related issues with their friends (70% vs. 65%, respectively; p = 0.01); being comfortable initiating talks with their friends about abortion-related issues (73% vs. 67%, respectively; p < 0.01); confident about their ability to help a friend find abortion services if needed (81% vs. 74%; p < 0.01); saying the words “vagina” and “penis” without embarrassment (18% vs. 11%; p < 0.01) and supporting a friend if she needed an abortion (88% vs. 83%; p = 0.02). On the other hand, more never-married women than ever-married women expected to be treated badly if they had an abortion (70% vs. 55%; p <0.01) and fewer imagined that health care workers would treat them kindly if they needed an abortion (37% vs. 54%, respectively; p < 0.01).

## Discussion

In this household-based survey conducted in Rupandehi district in Nepal, young (16–24 years of age) women reported having good access to information on reproductive health: most women had received information on sources and methods of contraception and abortion care in the past year. Furthermore, most women (88%) could name a method of abortion, most often medical abortion and, to a lesser extent, dilation and curettage. Few participants (7%) mentioned MVA as a possible method. Programmatic efforts should focus on increasing young women’s knowledge of MVA and equipping providers with the proper training and capacity to perform this safer alternative to dilation and curettage [26]. In Nepal, medication abortion is available at primary health centers while MVA services are accessible at higher levels of the health system. Improving women’s understanding of medication abortion regimens, and the optimal gestational age for accessing these services, could further improve reproductive health outcomes.

Participants denied having engaged in romantic activity, including sexual activity, outside of marriage. However, they were much more likely to report that their friends engaged in romantic relationships, spending time alone with a boy, kissing, and sexual contact outside of marriage. These findings suggest that social desirability bias might prevent women from admitting to having premarital experiences. This interpretation is consistent with a survey of students conducted at three colleges in Nepal, in which premarital sex was reported by 45% of men but only by 4% of women [27]. Similarly, a study of young factory workers in Kathmandu Valley found that 35% and 16% of unmarried males and females, respectively, reported being sexually experienced [20]. Finally, in a survey of male college students, 39% reported having had premarital sex [21]. Together with the literature, the present findings suggest that despite prevailing social norms, romantic and sexual activity among unmarried youth in Nepal, particularly in urban areas, is a reality.

Knowledge of Nepal’s abortion law was low: only 45% of women were even aware that abortion is legal in Nepal, and substantial proportions of women erroneously believed that a range of conditions (e.g., marital status, having a pregnancy result from a contraceptive failure or not wanting or not being able to afford a child) would affect the legal right to an abortion. This finding is consistent with previous research documenting low levels of awareness of the legal status of abortion [28-30], including an earlier assessment in the same district, which included reports of “medical halls” where women go for unsafe abortion [31]. The lack of awareness of legal status appears to result in women seeking unsafe abortion [32]. We found that ever-married women, regardless of their age, were consistently less informed than never-married women about the legality of abortion.

Women’s sources for reproductive health information also differed by marital status. Never-married women were more likely than ever-married women to report receiving contraception or abortion information in the past year from television, radio, or a friend or neighbor. In contrast, ever-married women were more likely than never-married to have obtained contraceptive or abortion information from a FCHV, health care provider or their husband. The important role that health care providers hold in disseminating information on contraception and abortion to all women should be emphasized and strengthened.

Although most (74%) of the young women in this survey reported feeling comfortable in talking to a health care provider about abortion-related issues, about 23% (with no difference found by marital status) did not think that they could decline sex if they wanted to. Furthermore, women reported being embarrassed by references to male and female genitals. This lack of self-efficacy regarding sex and fertility is a potential barrier to seeking proper reproductive health care.

Women’s perceptions of abortion-related factors also differed by their marital status. In general, never-married women tended to be more comfortable and supportive of abortion. They were more likely to talk about abortion topics with their friends and were more comfortable with these discussions than were married-women. They also were more likely to indicate that they would support a friend who needed an abortion or feel confident that they could help a friend obtain the procedure. However, never-married women expected to experience more negative responses from health care workers, family and friends if they were to have an abortion than were married-women. Furthermore, both never and ever-married women agreed that abortion among unmarried women was not accepted in their community (88% and 80%, respectively). Abortion among unmarried women in Nepal is strongly condemned, and providers have voiced concerns that the legalization of abortion is leading to an increase in utilization of services among unmarried women [33]. Both society, in general, and health care providers are more accepting of abortion among married women. For example, a qualitative study of abortion providers in Kathmandu Valley revealed that even though providers perceived abortion for sex selection to be unethical, they still empathized with married women who faced pressure to have a male offspring and thought that abortion for this purpose should be available for some women [34].

## Conclusions

Given the early mean age of childbearing among women in Nepal [35], sexual activity among adolescents clearly is common and underscores the importance of access to contraceptive and abortion care among youth. Less attention, though, has been directed to differences in the provision of information and services to unmarried women. This could be especially important in patriarchal societies, which could be expected to have strong norms against sexual agency of unmarried women. The present study demonstrated substantial differences in abortion information, experiences and perception by marital status and highlights the need for the provision of sexual and reproductive health care knowledge and services to all women regardless of marital status.

## Abbreviations

FCHV: Female community health volunteer
MVA: Manual vacuum aspiration
NHRC: Nepal Health Research Council
PPS: Probability proportional to size
SD: Standard deviation
US: United States
VDC: Village development committee
